# Metabotypes with properly functioning mitochondria and anti-inflammation predict extended productive life span in dairy cows

**DOI:** 10.1038/srep24642

**Published:** 2016-04-19

**Authors:** K. Huber, S. Dänicke, J. Rehage, H. Sauerwein, W. Otto, U. Rolle-Kampczyk, M. von Bergen

**Affiliations:** 1 Institute of Animal Science, University of Hohenheim, Germany; 2 Institute of Animal Nutrition, Federal Research Institute for Animal Health, Braunschweig, Germany; 3 Clinic for Cattle, University of Veterinary Medicine Hannover, Foundation, Germany; 4 Institute for Animal Science, University of Bonn, Germany; 5 Department of Proteomics, Helmholtz Centre for Environmental Research, UFZ, Leipzig, Germany; 6 Department of Metabolomics, Helmholtz Centre for Environmental Research, UFZ, Leipzig, Germany; 7 Department of Chemistry and Life Sciences, University of Aalborg, Denmark

## Abstract

The failure to adapt metabolism to the homeorhetic demands of lactation is considered as a main factor in reducing the productive life span of dairy cows. The so far defined markers of production performance and metabolic health in dairy cows do not predict the length of productive life span satisfyingly. This study aimed to identify novel pathways and biomarkers related to productive life in dairy cows by means of (targeted) metabolomics. In a longitudinal study from 42 days before up to 100 days after parturition, we identified metabolites such as long-chain acylcarnitines and biogenic amines associated with extended productive life spans. These metabolites are mainly secreted by the liver and depend on the functionality of hepatic mitochondria. The concentrations of biogenic amines and some acylcarnitines differed already before the onset of lactation thus indicating their predictive potential for continuation or early ending of productive life.

Dairy cows selected for high milk yield have an intrinsic high risk for metabolic disorders postpartum^1^,^2^,^3^. The adaptation to the onset of lactation requires a highly flexible metabolism with effective utilization of nutrients for fuelling milk production, maintenance and immune function. However, most modern dairy cows have very short production life spans due to the development of several, often severe diseases related to energetic imbalance and metabolic dysregulation. Characterization of dysregulated metabolic pathways and identification of early biomarkers for such dysregulation would be of great advantage. For dairy cows identified as being at a high risk for dysregulation, preventive interventions could be timely initiated at the level of the individual cow, e.g. by diet. In the long term, the phenotypic information about a low risk for dysregulation could be used in breeding to improve animal health and productive life span of dairy cows.

Classical markers such as non-esterified fatty acids (NEFA), beta-hydroxybutyrate (BHBA) and insulin concentrations are of low predictive value to identify cows which are not adequately able to actually match the genetically defined milk production performance. High lipid mobilization as reflected by high plasma NEFA concentrations is the most commonly accepted metabolic response, which is supposed to predict metabolic imbalance in dairy cows. However, high mobilizing cows are often just not metabolically dysregulated and cope very well with the metabolic changes provoked by parturition and the onset of lactation^4^,^5^. Thus, novel biomarkers of not yet considered pathways in the metabolism of the dairy cow need to be identified.

In human medicine, metabolomic approaches were used to identify patients/individuals with high risks to develop metabolic diseases and to detect genetic defects in specific pathways^6^,^7^. Metabolic phenotypes of patients could thereby be established and biomarkers for dysregulated pathways were identified. Besides a general, most often only semiquantitative profiling, there is also the opportunity of quantitative profiling using many targeted multiple reaction monitoring (MRM)-based mass spectrometric assays in a scheduled MRM setting. A commercialized and nowadays widely used selection of targeted quantifications is provided by Biocrates (Salzburg, Austria). The selection of quantified metabolites focuses on lipid metabolites, but covers also nearly all amino acids and many biogenic amines. Consequently, this metabolite panel has been proven to be useful in large-cohort studies, focusing on identifying novel biomarkers indicating human individuals at high risk for disease related to metabolic syndrome and diabetes type 2^8^. Recently, also in dairy research metabolomic approaches were performed in body fluids by GC-TOF/MS or triple quadrupole MS using the Biocrates metabolite panel^9^,^10^,^11^. Imhasly *et al*.^10^ studied the transition from gestation to early lactation but focused on hepatic lipidosis; the stage of hepatic lipidosis was confirmed by liver biopsy sampling in those cows. Hailemariam *et al*.^11^ studied the difference in 12 cows, from which six became ill during the periparturient period. In their study the time points −4, −1, 1, and 4 weeks in relation to parturition were analyzed. In contrast to those studies that were successfully defining molecular markers for evident diseases we stratified the animals by different criteria. In this study we aimed at identifying markers and (patho-) physiological processes in phenotypically healthy individuals that have a predictive value for later occurring health disturbances which may reduce the productive life span. Therefore we compared animals that were healthy for the entire lactation period *versus* those remaining healthy during the periparturient period from day −42 until day +100 but left productive life thereafter within the current lactation. We performed a targeted metabolomics approach in blood serum samples collected at different time points within the periparturient period of dairy cows to assess the dynamic changes in metabolite concentrations in plasma. The results of metabolic phenotyping were then linked to health status und length of productive life of these cows. Thus, the study aimed to describe a metabolic profile which may allow identification of a novel health metabotype predictive for metabolic flexibility and thereby, length of a cow’s productive life span.

## Results and Discussion

### Animal grouping

According to the cow’s history, the time of leaving productive life as grouping criterion revealed two groups. Group LE (= **L**eft productive life **E**arly) comprised 8 cows that were clinically healthy until day  +100, but left productive life within the current lactation due to various health and fertility problems whereupon it was decided to cull them. Group H (= **H**ealthy) consisted of 11 cows that were clinically healthy during the trial and finished the current lactation without any signs of clinical illness (details see Methods chapter).

### Characterization of phenotypes

Classical data describing the metabolic situation of dairy cows, including performance data such as dry matter intake (DMI), energy balance (EB), daily amount of fat corrected milk (FCM, kg/d) and 200 days milk amount (kg), were also assessed in the LE and H cows throughout the ante partum (ap) period and during lactation, respectively^12^. Imbalanced cows were commonly discussed to show excessive lipid mobilization with high plasma NEFA and BHBA, fatty liver and insulin resistance^1^.

However, DMI and EB of the 19 cows of groups LE and H were not significantly different (Table 1). Likewise, daily milk performance (LE: 35.5 ± 1.3 kg FCM/d, H: 33.7 ± 1.2 kg FCM/d) and 200 d lactation performance (LE: 6965 ± 484 kg, H: 7444 ± 296 kg) were similar in both groups. Plasma NEFA, BHBA and insulin concentrations showed the characteristic periparturient time course with an increase in NEFA and BHBA pp, while insulin decreased (Table 1). Again, no statistically significant difference was observed between LE and H cows. Obese cows over-conditioned in late pregnancy were discussed to be at high risk for metabolic disorders pp^13^. BFT, BCS and plasma leptin concentrations were therefore used to describe the initial situation regarding over-conditioning at day −42 of the cows used in this study. However, BFT (Table 1), BCS (LE: 3.26 ± 0.16, H: 3.32 ± 018) and leptin (LE: 8.57 ± 0.68 ng/ml, H: 10.9 ± 1.12 ng/ml) were not significantly different between LE and H groups. To define “obesity” in LE and H cows more accurately, liver triglyceride accumulation (LTAG), visceral adipose tissue (VAT) amount and plasma adiponectin concentration were determined throughout the periparturient period by ELISA and ultrasound measures, respectively. LTAG was not different in both groups (Table 1); however, H cows had higher amounts of VAT than LE cows throughout the periparturient period (Table 1). Consistently, since adiponectin is highly synthesized by VAT^14^, H cows also had higher adiponectin concentrations in plasma, especially around parturition (Table 1). Therefore, it can be suggested that an adequate amount of adiponectin-producing visceral adipose tissue was a physiological prerequisite in dairy cows to maintain metabolic balance throughout lactation. Adiponectin is an adipokine with insulin sensitizing effects, thereby contributing to metabolic health in humans^15^, and most likely also in cows^14^.

### Grouping and identification of grouping variables

**Figure 1:**
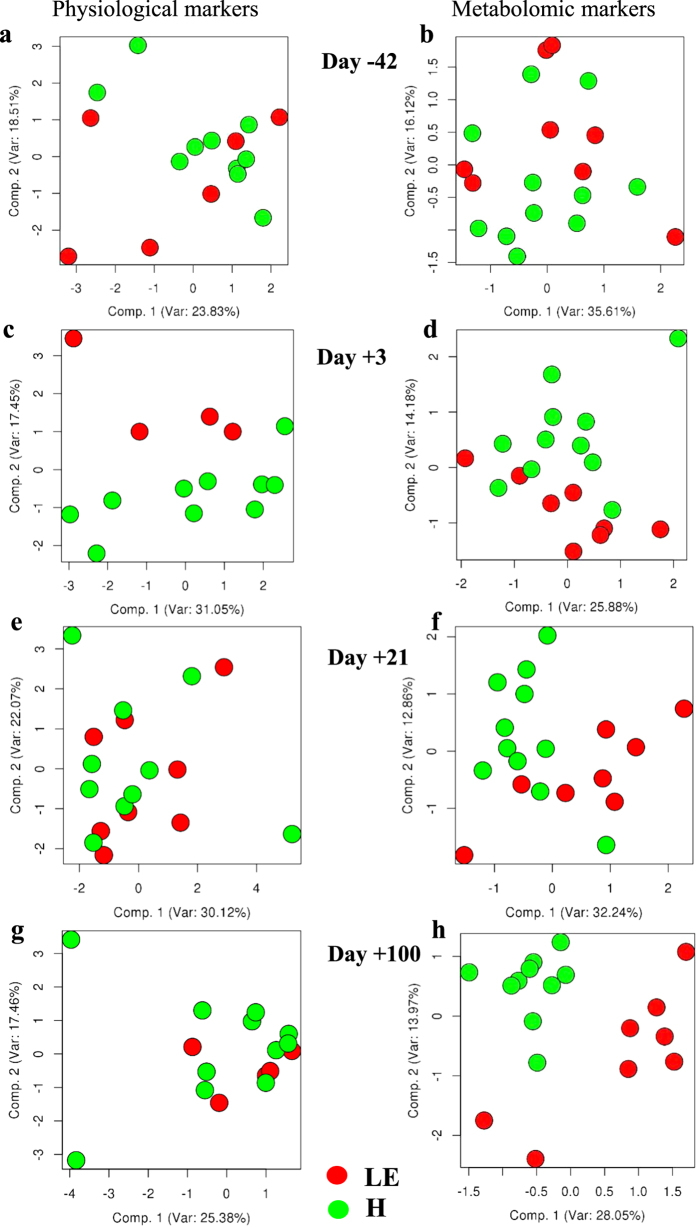
Exploratory data analysis to identify metabotypes. Principal component analyses (PCA) of physiological (dry matter intake, energy balance, glucose, non-esterified fatty acids, beta hydroxybutyrate, glycerol, insulin, liver triglycerides, visceral adipose tissue, back fat thickness) and metabolomics (acylcarnitines, proteinogenic and modified amino acids, glycerophospho- and sphingolipids, biogenic amines and hexoses) markers at days −42, +3, +21 and +100 related to calving. The two metabotypes, cows left productive life early (LE, red dots) and healthy cows (H, green dots) were clearly separated by metabolomics markers at day 21 and 100, while physiological markers revealed a clear separation at day +3 only.

**Figure 2:**
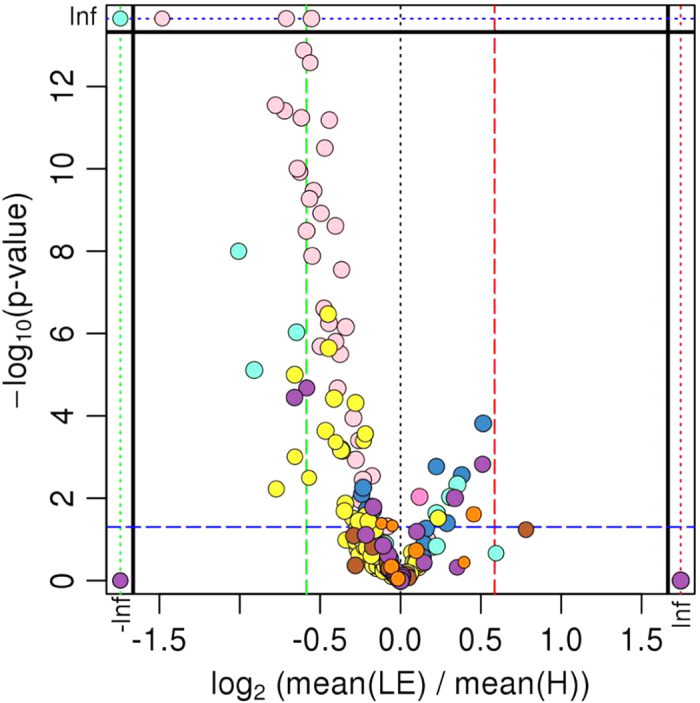
Visualization of changes in metabolite patterns by Volcano Plot. Statistical evaluation of group differences by t-test (visualized by Volcano Plot; it plots fold-changes versus significance on the x and y axes, respectively, for all metabolomic data) revealed significant upregulation (shift to the left) of certain metabolites in the healthy cow (H) group compared to the group of cows left productive life early (LE). Each colored dot represents one metabolite. Thus, the most-meaningful changes were identified. In a second evaluation step, these metabolites were tested by Two Way ANOVA for the factors group and time to identify metabolic markers associated with health and extended productive life (see Figures 3-5).

Considering physiological (DMI, EB, glucose, NEFA, BHBA, glycerol, insulin, LTAG, VAT, BFT) and metabolomics markers, at day −42, PCA of both did not reveal any separation of groups (Fig. 1a,b). Only at day +3 the PCA of physiological markers indicated a clear separation of the two different groups in the cohort (Fig. 1c), while PCA of metabolomics markers showed lower power to discriminate between the two groups (Fig. 1d). However, at day +21 (Fig. 1e,f) and more pronounced, at day +100 (Fig. 1g,h) the PCA of metabolomic markers indicated a clear separation of LE and H group, while physiological markers did not reveal any separation. The distribution of metabolites in the comparison of LE *versus* H revealed over all time points a significant up-regulation of certain metabolites in the H group (Fig. 2).

### Key metabolites indicating properly functioning mitochondria

**Figure 3:**
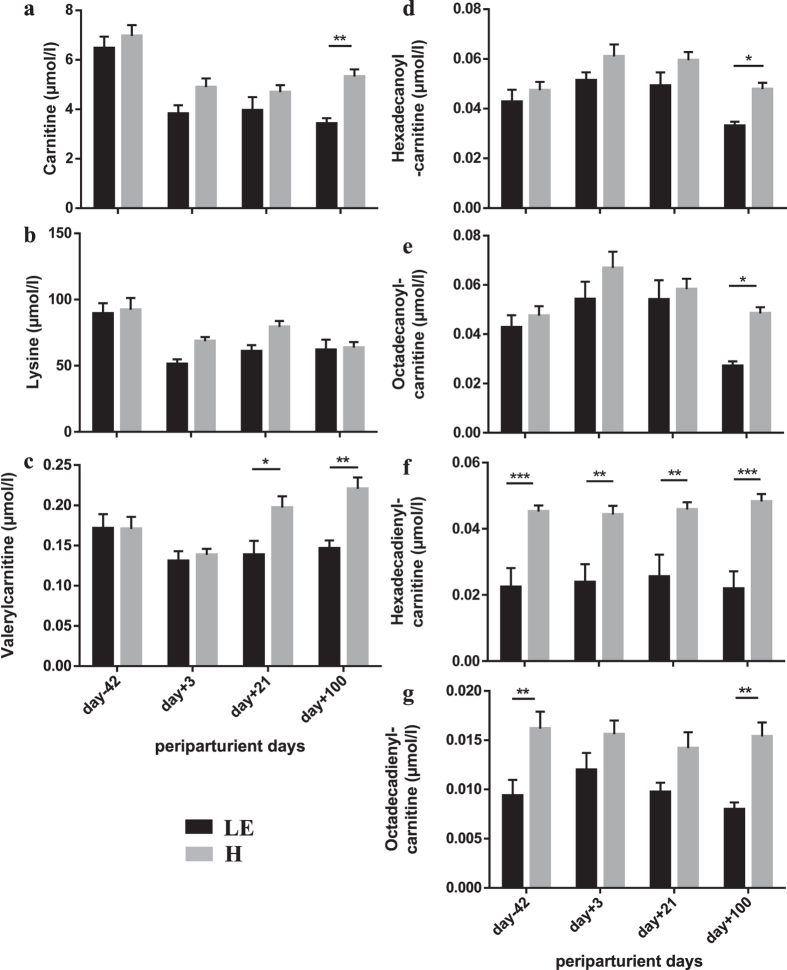
Carnitine and acylcarnitines in healthy (H) and left productive life early (LE) cows. Serum carnitine (**a**), lysine (**b**), valerylcarnitine (**c**), hexadecanoylcarnitine (**d**), octadecanoylcarnitine (**e**), hexadecadienylcarnitine (**f**) and octadecadienylcarnitine (**g**) concentrations in cows that left productive life early (LE, black bars, n = 8) or were healthy (H, grey bars, n = 10) as influenced by parturition and onset of lactation. Given were means ± SEM; *p < 0.05, **p < 0.01, ***p < 0.001. Results of repeated measures Two-Way ANOVA and Sidak’s multi comparison posttest (Graphpad.prism version 6.0) demonstrate effects of time and grouping and point out interactions between them (results see Table 2 below).

The capacity to utilize substrates, especially fatty acids, for generating of ATP in the respiratory chain requires effective oxidative pathways in mitochondria and is suggested to be an essential prerequisite for metabolic health. The carnitine and acylcarnitine pathway belongs to that capacity and was of particular importance for discriminating the LE *versus* the H group. Carnitine, synthesized from lysine and methionine in a PPARα-dependent manner in liver, brain and kidney or ingested with the diet, is essential for mitochondrial fatty acid oxidation and improves glucose homeostasis^16^. H group cows had higher carnitine concentrations throughout the periparturient period, which was especially obvious at day 100 (Fig. 3a). Concomitantly, H cows had higher plasma lysine concentrations indicating better educt availability for carnitine synthesis (Fig. 3b). Carnitine was also identified as an outstanding disease biomarker in dairy cows developing sickness already during the perparturient period^11^; however, these cows suffering from different production diseases at the time of examination had higher carnitine concentrations in serum within the transition period. This was suggested to be the consequence of tissue cell death and organ dysfunction leading to release of carnitine and acylcarnitines into serum. The higher carnitine concentrations in H group may support effective mitochondrial acylcarnitine formation by carnitine palmitoyl transferase 1 (CPT1), thereby reducing oxidative stress and accumulation of reactive oxygen species.

**Figure 4:**
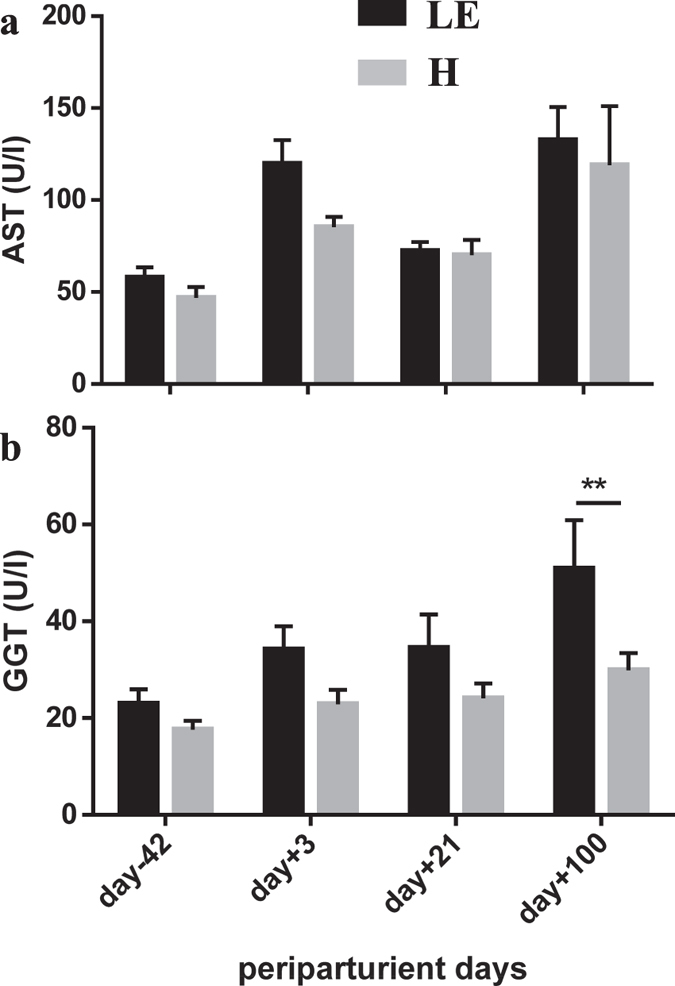
Liver enzymes in healthy (H) and left productive life early (LE) cows. Serum aspartate transaminase (AST) (**a**) and gamma glutamyl transferase (GGT) (**b**) activity in cows that left productive life early (LE, black bars, n = 8) or were healthy (H, grey bars, n = 11) as influenced by parturition and onset of lactation. Given were means ± SEM; **p < 0.01. Results of Two-Way ANOVA and Sidak’s multi comparison posttest (Graphpad.prism version 6.0) demonstrate effects of time and grouping and point out interactions between them (results see Table 3 below).

Accordingly, major changes were found in serum concentrations of valerylcarnitine (Fig. 3c), hexadecanoylcarnitine (Fig. 3d), octadecanoylcarnitine (Fig. 3e), hexadecadienylcarnitine (Fig. 3f) and octadecadienylcarnitine (Fig. 3g). The concentrations of these acylcarnitines were higher in H cows compared to LE cows throughout the periparturient period and at any specific time points. The acylcarnitines with double bounds in the fatty acids were already lower ante partum in LE cows indicating hexadecadienylcarnitine and octadecadienylcarnitine as predictive markers relevant for the risk of developing metabolic dysregulation and for leaving productive life early. The biological impact of these differences cannot be defined yet and has to be assessed by further studies. However, these findings led to the hypothesis that higher levels of acylcarnitines in serum of H cows may reflect their capacity to adapt mitochondrial functions properly to the metabolic situation postpartum. Probably hepatic mitochondria are primarily affected, because the liver is discussed to be the main source of serum acylcarnitines^16^. This could also be suggested for the dairy cow, especially during the transition from pregnancy to lactation when most of ATP is produced by fatty acid oxidation in the liver. However, it was unlikely that the greater concentrations of carnitine and acylcarnitine in serum from H cows were associated with enhanced liver cell death and organ dysfunction. While aspartate transaminase was similar in H and LE cows (Fig. 4a), gamma glutamyl transferase, as a well-known marker for hepatocyte integrity of dairy cows^17^, was significantly lower in H cows compared to LE cows indicating a less affected liver function in H cows (Fig. 4b). Thus, higher levels of acylcarnitines in serum of H cows appeared to be beneficial. It is suggested that this reflects a greater ability of hepatocytes to release any surplus of carnitine and acylcarnitines from the mitochondria to avoid mitochondria damage. Specific transporters existed in membranes of cells and mitochondria for the uptake or release of carnitine (SLC22A5, OCTN2; novel organic cation transporter 2) and of acylcarnitines (SLC16A9; MCT9, monocarboxylate transporter 9)^16^. The OCTN2 is known to be upregulated in livers of early lactating cows^18^ and to be stimulated by PPARα and proinflammatory cytokines in bovine kidney cells^19^. Since hepatocytes are highly capable of synthesizing carnitine, especially in early lactation^16^,^18^, an uptake of carnitine appeared to be biologically unnecessary. This indicates an important role of OCTN2 for carnitine export from liver for the use in other peripheral tissues. Information about the existence and regulation of MCT9 transporters in bovine liver is not available so far.

To summarize, in addition to the fact that the liver is also the main source for serum acylcarnitines^16^, carnitine ester concentrations in bovine liver tissues were very low postpartum^18^. These findings support the above mentioned hypothesis that an increased hepatic export of carnitine and acylcarnitines is an important adaptive process in early lactating dairy cows. Thus, the liver may allocate the excess of carnitine and acylcarnitines to other peripheral tissues including mammary gland, and thereby support effective oxidative energy production in peripheral tissues and avoid liver cell damage by accumulation of fatty acid degradation intermediates.

### Key metabolites indicating anti-inflammation

Since intracellular accumulation of derivatives of lipid metabolism and deprivation of carnitine caused oxidative stress, mitochondrial dysfunction, insulin resistance and increased pro-inflammatory cytokine expression (reviewed by^20^), it was expected that a high hepatic capacity for release of lipid metabolism intermediates such as acylcarnitines into the blood was connected with less inflammation in H cows. In confirmation, metabolites indicating inflammation, oxidative stress and metabolic aging were lower in H cows compared to LE cows. These metabolite patterns belonging to the tryptophan (Trp)/kynurenine (Kyn) pathway and to the class of biogenic amines indicated an improved metabolic balance in H cows.

**Figure 5:**
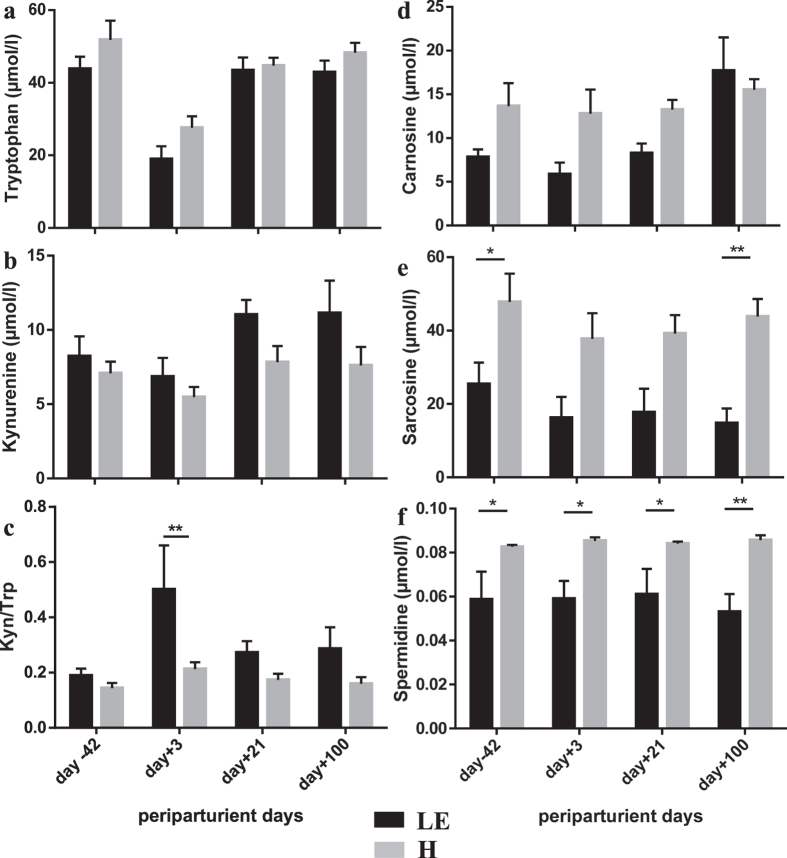
Tryptophan, kynurenine and biogenic amines in healthy (H) and left productive life early (LE) cows. Serum tryptophan (**a**), kynurenine (**b**), kynurenine/tryptophan ratio (Kyn/Trp) (**c**), carnosine (**d**), sarcosine (**e**) and spermidine (**f**) in cows that left productive life early (LE, black bars, n = 7–8) or were healthy (H, grey bars, n = 8–10) as influenced by parturition and onset of lactation. Given were means ± SEM; *p < 0.05, **p < 0.01. Results of Two-Way ANOVA and Sidak’s multi comparison posttest (Graphpad.prism version 6.0) demonstrate effects of time and grouping and point out interactions between them (results see Table 4 below).

The amino acid Trp was significantly higher in H cows, while its degradation product, Kyn was lower demonstrating less activated Trp degradation (Fig. 5a,b). Consequently, the Kyn/Trp ratio, an indicator of low-grade chronic inflammation in humans^21^, was lower in H cows (Fig. 5c). In contrast, LE cows had higher Kyn/Trp ratios throughout the periparturient period with a massively higher value shortly after parturition. This enhanced Trp degradation might reflect the inability of LE cows to avoid metabolic imbalance in a very early period of production, most likely based on a low capacity to protect mitochondria by an effective release of acylcarnitines. Mitochondrial dysfunction caused secretion of pro-inflammatory cytokines, which stimulated Trp degradation to Kyn by the indoleamine 2,3 diaminooxidase (IDO-1)^22^. This new pathway may indicate low-grade chronic inflammation in LE cows, which however did not lead to clinically obvious diseases within the experimental period, but compromised the length of their productive life span. Furthermore, Kyn and its degradation intermediates were involved in the development of insulin resistance, especially in obese humans^22^, in the process of inflammageing^23^ and also in modulation of immune cell function in humans and cows^24^,^25^ - pathways which might also be affected in high-yielding dairy cows. Thus, the metabolites of the Kyn/Trp pathway should in the future also be considered as molecules discriminating balanced and non-balanced metabolic phenotypes in dairy cows.

Inflammation and mitochondrial dysfunction also point to pathways underlying the metabolic imbalance in LE cows as indicated by the Kyn/Trp ratio and less carnitine and acylcarnitines in serum from day −42 before until day +100 after calving. In confirmation, the concentrations of anti-inflammatory and anti-oxidative biogenic amines such as carnosine (Fig. 5d), sarcosine (Fig. 5e) and spermidine (Fig. 5f) were significantly lower in LE cows compared to H cows. While sarcosine and spermidine were constantly lower from day −42 before until day 100 after calving without any time-related effect, carnosine showed highest values at day 100 without any difference between H and LE cows. However, from day −42 up to day 21 the concentration of this biogenic amine was significantly lower in LE cows.

In general, common effects of biogenic amines such as carnosine, sarcosine and spermidine are anti-inflammatory, anti-oxidative and anti-ageing^26^. Carnosine (β-alanyl-L-histidine) belongs to the non-protein nitrogen-containing compounds and is primarily synthesized by muscle cells^27^. It plays a role in cellular pH buffering, in anti-oxidative protection of the cell by scavenging reactive oxygen species (ROS) and in cellular defense against formation of advanced glycoxidation and lipoxidation end-products^27^,^28^. The concentration in serum may reflect intracellular concentrations; the LE cows may thus have less benefit of carnosine especially from day −42 up to day 21 than the H cows. This could indicate less protection against oxidative damage in the first three weeks of lactation. The dramatically higher values of carnosine at day 100 in LE cows might reflect muscle cell degradation and thereby increased carnosine release into serum. Aspartate transaminase activity, as a marker for muscle damage, was higher at day 100 in LE cows but this was also the case in H cows (Fig. 4a). Thus, this result is difficult to interpret and needs further examination. As day 100 was often noticeable in terms of significant differences in metabolite concentrations (carnitine, C5, C16, C18, gamma glutamyl transferase) between LE and H cows, those and the increase in carnosine might demonstrate initiation of metabolic dysregulation, which lead to leaving productive life early.

Sarcosine (N-methylglycine) is an intermediate product from glycine metabolism and is produced by all body cells. As dietary supplements, sarcosine promotes neuroprotection^29^ and its precursor, betaine, increases lean mass, decreases body fat accretion, enhances insulin sensitivity and stimulates growth hormone secretion (reviewed by^30^). Furthermore, sarcosine can serve as methyl donor, thereby causing epigenetic changes by methylation of DNA and providing methyl groups for carnitine synthesis^30^.

Spermidine, a polycationic aliphatic amine (polyamine), is synthesized by somatic cells but also by gut microbiota. Its concentrations in the body decrease during the ageing process indicating a linkage to longevity in mice; and it is suggested that polyamines increase longevity by decreasing low-grade inflammation in the gut and the body^26^. The capability of spermidine to modulate autophagy, a mechanism responsible for regeneration of cellular components and organelles, is discussed to be the major underlying pathway for longevity and other beneficial effects of spermidine^31^. Studies in dairy cows about spermidine effects are not existent to the best of our knowledge. Since polyamines can be also modulated by diet directly or indirectly by dietary modulation of microbial population and its polyamine production, these metabolites are not only good candidates as biomarkers for metabolic balance but also a target for nutriceutical approaches to improve anti-inflammation and to extend productive lifetime in dairy cows.

## Conclusions

By considering metabolomic data in blood of periparturient dairy cows we suggest the carnitine and acylcarnitine production and the capacity for its secretion from hepatocytes as a potentially crucial event in supporting a healthy metabotype in the periparturient period. Even if the sample size is limited the identified dysregulated pathways in the LE group appeared to be fundamental and evolutionary highly conserved pathways with a strong impact for balanced metabolic performance also in other species. Extrapolation of these findings to dairy cows world-wide is difficult; however, this study facilitates creation of new hypotheses on metabolic dysregulation and energetic imbalance in dairy cows which are not based on detrimental effects of excessive lipid mobilization but on consequences of less effective mitochondrial function. Finally, potentially signaling metabolites have to be validated as biomarkers in large-cohort studies to be used for management and feeding programs in dairy herds for health protection of individual cows at high risk. Furthermore, these potential new biomarkers, if proven, could support genetic selection of sires and dams in dairy breeding to increase the numbers of cows with inherited traits for an “extended productive life span” metabotype.

## Methods

### Animals

All animal experiments were performed in accordance with the German Animal Welfare Act, and approved by the Lower Saxony State Office for Consumer Protection and Food Safety (LAVES), Oldenburg, Germany.

Multiparous Holstein dairy cows of an experimental herd at the Federal Research Facility for Farm Animals (FLI) in Braunschweig, Germany, in their second to fourth lactation were used to perform a feeding trial with variations in concentrate feeding ante partum (ap) and niacin supplementation ap and up to day 24 pp; the experiment lasted from day −42 (expected to calving) ap up to day +100 postpartum (pp) in total. Background data such as experimental design, feeding regimen, methods and animal data were already published elsewhere^10^. The feeding regimen did not affect measured animal performance data (dry matter intake (DMI), energy balance (EB), body condition score (BCS), back fat thickness (BFT), milk yield) and classical metabolic variables (NEFA, BHBA, insulin, liver enzymes) in blood and tissues^10^. However, inter-individual variation in all measured variables over time was indicating considerable different individual metabolic responses to the onset of lactation. Thus, data were re-analyzed by using the following criteria.

The individual history of the 20 cows - analyzed in-depth in former studies^12^ - was followed up according to the herd recordings regarding the time of leaving productive life.

For identifying new metabotype-grouping variables, serum samples of 19 cows (one sample was lost due to technical reasons) were used for a targeted metabolomic approach. Serum samples were selected from day −42, −10 (to expected calving), +3, +21 and +100 for analysis. The reasons why cows left productive life early varied to some degree. Main reasons were metabolic disorders, related inflammatory production diseases and infertility reflected by prolonged first estrus interval pp and low conception rates. Other features from the cows’ histories such as genetic origin (fathers), feeding regimen, age, lactation numbers or other aspects were randomly distributed between the three groups indicating that the ability for metabolic and energetic balance and thereby, health and productivity, was a major determinant for grouping.

### Metabolomics

For quantification of metabolites, a targeted, standardized and quality controlled metabolic phenotyping was performed based on LC/MS-MS analysis. The metabolome analyses were carried out using the AbsoluteIDQ^®^ p180 Kit (Biocrates Life Science AG, Innsbruck, Austria). The kit identifies and quantifies 188 metabolites from 5 compound classes, namely acyl carnitines^40^, proteinogenic and modified amino acids^19^, glycerophospho- and sphingolipids (76 phosphatidylcholines, 14 lysophosphatidylcholines, 15 sphingomyelines), biogenic amines^19^ and hexoses (for details see www.biocrates.com/products/research-products/absoluteidq-p180-kit). All reagents used in the processing and analysis were of LC-MS grade. From the serum samples 10 μL were mixed with isotopically labeled internal standard in a multititer plate and dried under nitrogen (nitrogen evaporator 96 well plate, VLM GmbH, Bielefeld, Germany). Afterwards the metabolites were derivatized with phenylisothiocyanate (PITC) 5% for 20 min at room temperature and subsequently dried for 30 min under nitrogen flow. For extraction first 300 μL of extraction solvent (5 mM ammonium acetate in methanol) were added and incubated with shaking at 450 rpm (Thermomixer comfort Eppendorf, Hamburg, Germany) for 30 min at room temperature followed by filtration by centrifugation (Sigma 2-16 k, Taufkirchen, Germany) for 2 min at 500 × g. Subsequently 200 μL were removed from the filtrate, transferred to a fresh multititer deepwell plate and diluted with 200 μL of water (LC/MS grade) for LC-MS analysis of biogenic amines and amino acids. To the remaining 100 μL from the filtrate 500 μl of MS running solvent were added for flow injection analysis-MS/MS measurements (FIA-MS/MS). Both types of measurements were performed on a QTRAP mass spectrometer applying electrospray ionization (ESI) (ABI Sciex API5500Q-TRAP). The MS was coupled to an UPLC (Waters Acquity, Waters Corporation, Milford, USA). In case of LC-MS the metabolites were separated by an hyphenated reverse phase column (Agilent, Zorbax Eclipse XDB C18, 3.0 × 100 mm, 3.5 μm, Agilent Waldbronn, Germany) preceded with a precolumn (Security Guard, Phenomenex, C18, 4 × 3 mm; Phenomenex, Aschaffenburg, Germany) applying a gradient of solvent A (formic acid 0.2% in water) and solvent B (formic acid 0.2% in acetonitrile) over 7.3 min (0.5 min 0% B, 5 min 70% B, 0.3 min 70% B, 2 min 0% B) at a flow rate of 500 μl/min. Oven temperature was 50 °C. For LC-MS analysis 10 μl and for FIA 2 times 20 μl for measurements in positive and negative mode were subjected. Identification and quantification were achieved by multi reaction monitoring (MRM) standardized by applying spiked-in isotopically labelled standards in positive and negative mode, respectively. For calibration a calibrator mix consisting of 7 different concentrations was used. Quality controls were included for 3 different concentration levels. For FIA an isocratic method was used (100% organic running solvent) with varying flow conditions (0 min, 30 μL/min; 1.6 min 30 μL/min; 2.4 min, 200 μL/min; 2.8 min, 200 μL/min; 3 min 30 μL/min), and the MS settings were as follows: scan time 0.5 s, IS voltage for positive mode 5500 V, for negative mode −45000 V, nitrogen as collision gas medium, source temperature 200 °C; the according parameters for LC-MS were scan time 0.5 s, source temperature 500 °C, nitrogen as collision gas medium).The integrated MetIDQ software (Biocrates, Innsbruck, Austria) streamlines data analysis by automated calculation of metabolite concentrations providing quality measures and quantification.

### Bioinformatic evaluation

Metabolomic data of cows grouped according to their history were evaluated by discriminant analysis to verify grouping using the free software package R (www.r-project.org). Furthermore, metabolomes of cows grouped according to their history were compared at each time point by Vulcano Plots (R software). This method was used to visualize differences between the two groups and to identify the metabolites which vary significantly between the groups regarding their fold-change. The principal component analysis (PCA) of the samples was performed with the princomp function of GNU R. The analyte concentrations were normalized by calculation of the standard score. Cows grouped according to their history were tested for significance of difference in those highly varying metabolites by using repeated measures (rm) Two Way ANOVA and Sidak’s multi comparison posttest (Graphpad.prism version 6.0). Values were given as means ± SEM, LE group n = 8, H group n = 11, if not otherwise stated in the figure legends. Full sample size could often not be considered for rm Two Way ANOVA due to single missing values at single time points. A p-value < 0.05 was considered to be significant.

## Additional Information

**How to cite this article**: Huber, K. *et al*. Metabotypes with properly functioning mitochondria and anti-inflammation predict extended productive life span in dairy cows. *Sci. Rep.* **6**, 24642; doi: 10.1038/srep24642 (2016).

## Figures and Tables

**Table 1:** Animal performance and condition data (means ± SEM, LE n = 6–8; H n = 8–11).

Variables	Day^1^	Unit	Groups		Two Way ANOVA^2^		
			LE	H	day (d)	group (g)	d × g
Dry matter intake	−42	kg/d	16.2 ± 0.8	15.7 ± 0.8	p < 0.0001	n.s.	n.s.
	+3		11.2 ± 0.9	12.1 ± 0.7			
	+21		17.9 ± 1.1	17.5 ± 0.6			
	+100		23.2 ± 0.5	23.4 ± 0.6			
Energy balance	−42	MJ/d	68.2 ± 9.3	58.5 ± 6.9	p < 0.0001	n.s.	n.s.
	+3		−98.3 ± 13.9	−87.3 ± 9.7			
	+21		−43.8 ± 7.7	−48.5 ± 3.9			
	+100		3.2 ± 4.9	6.9 ± 4.8			
Back fat thickness	−42	mm	7.6 ± 06	10.4 ± 1.6	p = 0.0002	n.s.	n.s.
	+3		13.7 ± 1.5	13.3 ± 1.8			
	+21		11.3 ± 1.8	11.8 ± 1.2			
	+100		5.9 ± 0.3	7.8 ± 1.2			
Non-esterified fatty acids	−42	mmol/l	0.22 ± 0.04	0.15 ± 0.01	p < 0.0001	n.s.	n.s.
	+3		0.71 ± 0.15	0.73 ± 0.12			
	+21		0.47 ± 0.06	0.40 ± 0.04			
	+100		0.17 ± 0.02	0.19 ± 0.02			
Beta-hydroxy-butyrate	−42	mmol/l	0.57 ± 0.07	0.67 ± 0.05	n.s.	n.s.	n.s.
	+3		0.82 ± 0.11	0.87 ± 0.13			
	+21		0.88 ± 0.18	1.12 ± 0.38			
	+100		0.75 ± 0.07	0.72 ± 0.04			
Insulin	−42	mU/l	38.4 ± 9.52	25.1 ± 5.82	p = 0.0061	n.s.	n.s.
	+3		14.6 ± 3.22	18.2 ± 3.69			
	+21		14.4 ± 1.46	18.7 ± 2.89			
	+100		20.8 ± 3.66	20.8 ± 3.01			
Liver triglycerides	−42	nmol/mg	1.5 ± 0.4	0.9 ± 0.1	p < 0.0001	n.s.	n.s.
	+3		25.7 ± 4.4	21.2 ± 5.5			
	+21		35.5 ± 6.3	35.4 ± 11			
	+100		3.5 ± 2.8	2.1 ± 1.5			
Visceral adipose tissue	−42	kg	34.0 ± 4.9	45.6 ± 4.0	p < 0.0001	p = 0.0003	n.s.
	+3		39.9 ± 6.6	57.1 ± 2.5*			
	+21		29.5 ± 5.5	40.0 ± 3.9			
	+100		22.3 ± 3.2	32.0 ± 4.2			
Adiponectin	−42	μg/ml	27.8 ± 1.9	36.1 ± 4.8	p = 0.0003	p = 0.0428	n.s.
	+3		19.6 ± 2.4	22.8 ± 1.0			
	+21		25.2 ± 1.7	30.1 ± 0.8			
	+100		28.0 ± 2.3	29.2 ± 1.1			

^1^Days related to parturition; +ante partum, − postpartum.

^2^Two Way ANOVA for factor day, group and their interaction was performed, and Sidak’s multiple comparison post test (differences between LE and H indicated by *) was used (Graphpad.prism Version 6.0).

**Table 2:** Statistical analysis of results demonstrated in Fig. 3.

Variable	Two Way ANOVA		
	day (d)	group (g)	d × g
Carnitine	p < 0.0001	p = 0.0178	p = 0.0967
Lysine	p < 0.0001	p = 0.0376	n.s.
Valerylcarnitine	p = 0.0022	p = 0.0131	p = 0.0078
Hexadecanoylcarnitine	p = 0.0001	p = 0.0065	n.s.
Octadecanoylcarnitine	p = 0.0001	p = 0.0141	n.s.
Hexadecadienylcarnitine	n.s.	p = 0.0004	n.s.
Octadecadienylcarnitine	n.s.	p < 0.0001	n.s.

n.s. non significant.

**Table 3:** Statistical analysis of results demonstrated in Fig. 4.

Variable	**Two Way ANOVA**		
	day (d)	group (g)	d × g
Aspartate transaminase	p < 0.0001	n.s.	n.s.
Gamma glutamyl transferase	p = 0.0007	p = 0.0041	n.s.

n.s. non significant.

**Table 4:** Statistical analysis of results demonstrated in Fig. 5.

Variable	Two Way ANOVA		
	day (d)	group (g)	d × g
Tryptophan (TRP)	p < 0.0001	p = 0.0413	n.s.
Kynurenine (Kyn)	p = 0.0008	n.s.	n.s.
Kyn/Trp ratio	p = 0.0072	p = 0.0197	n.s.
Carnosine	p = 0.0051	p = 0.0115	n.s.
Sarcosine	n.s.	p < 0.0001	n.s.
Spermidine	n.s.	p < 0.0001	n.s.

n.s. non significant.
